# Performance Comparison of Advanced Ceramic Cladding Approaches via Solid-State and Traditional Welding Processes: A Review

**DOI:** 10.3390/ma13245805

**Published:** 2020-12-19

**Authors:** Senthil Kumaran Selvaraj, Kathiravan Srinivasan, Jainendra Deshmukh, Darshit Agrawal, Sailam Mungilwar, Rucha Jagtap, Yuh-Chung Hu

**Affiliations:** 1 Department of Manufacturing Engineering, School of Mechanical Engineering, Vellore Institute of Technology, Vellore 632 014, Tamil Nadu, India; senthilkumaran.s@vit.ac.in (S.K.S.); jainendra.deshmukh2018@vitstudent.ac.in (J.D.); darshit.agrawal2018@vitstudent.ac.in (D.A.); govind.mungilwar2018@vitstudent.ac.in (S.M.); ruchaumesh.jagtap2018@vitstudent.ac.in (R.J.); 2 School of Information Technology and Engineering, Vellore Institute of Technology (VIT), Vellore 632 014, Tamil Nadu, India; kathiravan.srinivasan@vit.ac.in; 3 Department of Mechanical and Electromechanical Engineering, National Ilan University, Yilan City 26047, Yilan County, Taiwan

**Keywords:** solid-state welding, fusion, ceramics, cladding, hardness, wear resistance

## Abstract

Ceramic coating has applications in enhancing the material’s properties and can significantly improve the material’s usability in varied temperatures and adverse operating conditions and widen its applicability scope. It can add to the various properties such as wear resistance, high-temperature degradation, thermal conductivity, material toughness, tensile strength, corrosion resistance, friction reduction, electric insulation, and the lifespan of the material. Various techniques have been suggested and implemented to achieve ceramic coating on a metal surface, each having their respective advantages and disadvantages. Hence, they can be distinguished for their applicability in different places. The bonding mechanism of metal particle systems has been researched to date, but there are still certain uncertainties regarding the ceramic particle system because of the dissimilarities in properties. The paper aims to profoundly investigate the various coating technologies available through welding processes and do a comparative study through numerical analysis and experimental results on the properties of coatings obtained from two broad categories of welding—solid-state and traditional/fusion processes. It was found that the solid-state processes in which the temperature remained well below the fusion temperatures overcame the mismatch in property and produced reliable coatings with enhanced mechanical properties.

## 1. Introduction

The modern era demands more complex operations in hostile environments while giving maximum efficiency at the same time. The judicious use of materials becomes an utmost priority to ensure fail-safe operation in such unfavorable conditions. The materials are hence coated with protective coatings to provide resistance to such an environment. Ceramic materials have found widespread applications in the field of coating and cladding due to its excellent properties. Though they cannot replace metals, they offer enhancement of properties such as excellent resistance to corrosion, wear, abrasion, degradation at high temperatures, etc. It also has many applications when different properties are required in different regions within the same material for a specific purpose [1]. It gives the user his freedom to choose a material based on the purpose which could easily be protected with a layer of ceramic coating without changing any design or structure considerations.

### Applications

**Figure 1:**
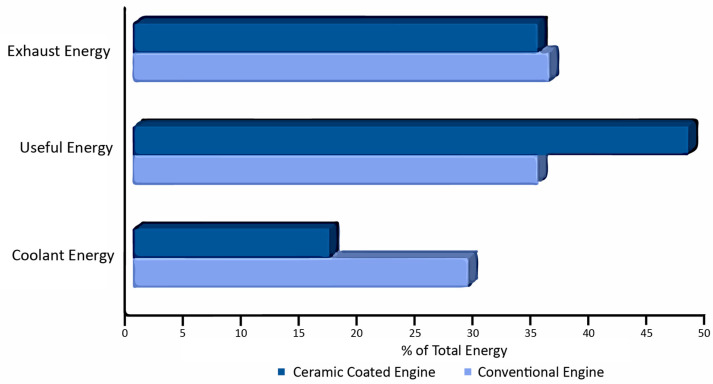
Property enhancement on the application of ceramic coatings in internal combustion engines.

**Figure 2:**
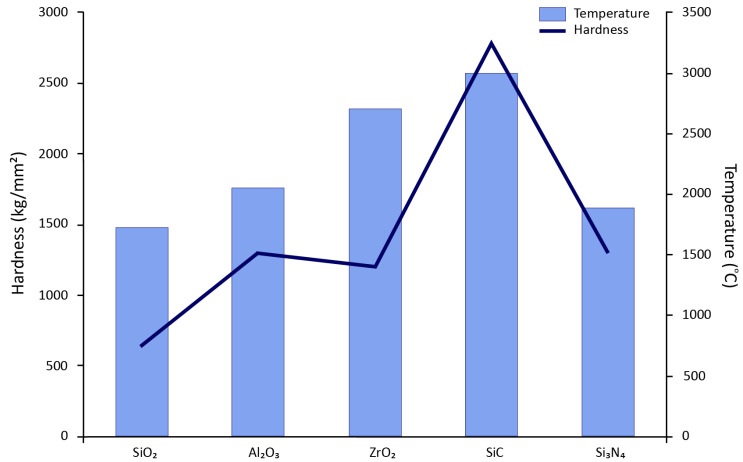
Property comparison of advanced ceramic coatings.

They find their application in process improvement, free-standing structures, and reclamation coatings. A broad application can be found in the automotive industry due to its low thermal conductivity and high thermal resistance property [2]. The combustion chambers of engines can be coated with ceramics to prevent the conduction of internal heat to the outside environment, thus increasing the efficiency of the combustion cycle [3,4]. Further, this can significantly reduce fuel [5,6] and can prove an excellent potential for eco-friendly vehicles. Moreover, this can also help in engine cooling. A significant portion of heat would remain inside the combustion chamber and hence can substitute the engine cooling system in the future. A comparative analysis is shown in Figure 1. The modernization of technology has brought forward the concept of advanced ceramic coatings with additional properties to enhance the substrate and its range of applicability. Some of the properties have been discussed in Figure 2.

It is widely used as a thermal barrier coating (TBC) due to its exceptional thermo-mechanical properties compared to metals. They find their use in gas turbine engines, power generation systems, and even marine propulsions where insulation is required from the hot steam [7]. They can efficiently operate at an elevated temperature beyond the fusion point of base metals/alloys and give them broad practicality in extreme conditions [8]. Moreover, they are usually required to last for a prolonged period under repetitive and extreme usage. However, it has some limitations and disadvantages to its domain of applicability in real-world conditions. There is a massive mismatch in metals and ceramics’ properties, the most important and influential in thermal expansion. It can lead to unequal expansion on experiencing temperature differences and can lead to cracks, which can propagate and cause failure.

They are also used as corrosion protection coatings in various industries. Most of the industries suffer from humid, extreme temperatures and other adverse working conditions. Ceramic materials are being used as a coating material, and they are cladded along with other layers to protect the base metal from corrosion. The introduction of nanotechnology in this field promotes easy and efficient coating mechanisms which can widely be used for their excellent corrosion resistant properties [9,10].

Besides their thermal and corrosion-resistant applications, stiffness and strength are required for ceramic coatings. Ceramics bear high specific strength and toughness, enhancing the wear resistance properties of raw materials [11]. These capabilities can be used in machining tools and other areas that suffer from excessive and repetitive wear and experience heavy loads for a shorter period [12].

Welding provides versatile methods for efficient and robust ceramic cladding with the metal/alloy substrates. There are various traditional methods such as thermal spraying, gas tungsten arc welding, gas metal arc welding, shielded metal arc welding, etc. Several non-traditional methods are plasma arc, friction stir, laser beam, etc. [13]. However, due to the difference in properties between metals and ceramics, the major is the thermal expansion, leading to the development of residual stresses during cladding, including melting and solidification phases, and causes cracks that easily propagate the degradation of the coating. Hence, only a few processes are successful in cladding these particles over the base material. When it comes to technological advancements, the primary concerns remain time and quality. This paper discusses the various processes suitable for cladding ceramics and performs a comparative analysis to conclude the best viable solution.

## 2. Literature Study

**Figure 3:**
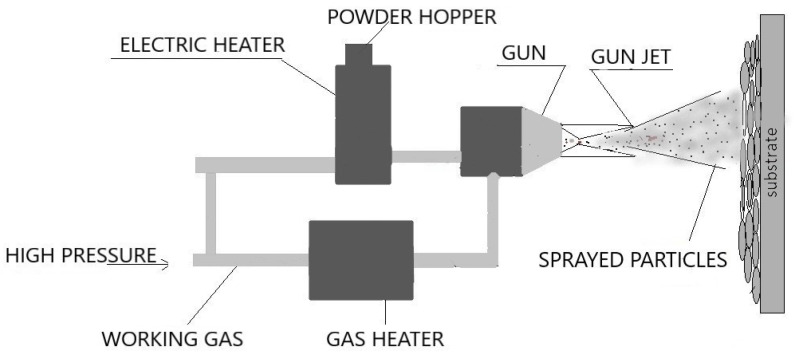
Cold spraying—a solid-state process.

Numerous research and discussions have led to developing viable solutions for coating the different elements together, especially the ceramic-metal system due to their wide range of applicability and excellent properties. Various methods have been investigated and results published based on their post-coating microstructures and mechanical properties. The broad categories can be listed down to solid-state welding and traditional welding processes, mainly using fusion methods. Fusion Welding Processes typically use heat for the coalescence of surfaces of the workpieces. Heat can be supplied by various sources such as laser, electric arc, gas flame, electrical resistance, etc., which defines the type of welding processes and significantly affects the microstructural and mechanical properties of the obtained weld. The solid-state process, on the other hand, produces coalescence below the fusion temperatures of the materials. Therefore, this process does not have to account for the property mismatch, which is the main advantage for joining dissimilar materials. The pressure is a variable factor and may or may not be applied depending upon the required conditions. An example of a solid-state process is shown in Figure 3.

The studies have also explored the respective modification and solutions to a particular method’s imperfections/defects. Still, mostly only a single or a restricted set of methods are discussed to comparatively analyze them based on their properties. No study has been conducted to provide the best method out of the two for addressing this issue. It is still uncertain as to which method to prefer both qualitatively and quantitatively. This paper aims to study all the possible methods available under the above two broad categories for cladding ceramic particles and, based on their post-cladding results, comparatively analyze and suggest the best-suited method for the specific purpose.

### 2.1. Processes and Sub-Systems for Ceramic Coating

#### 2.1.1. Gas Tungsten Arc Welding

Gas Tungsten Arc Welding (GTAW) can be utilized for cladding processes due to its merits of less solidification time and a small and concentrated heat-affected zone (HAZ), which produces fine microstructures resulting in a metallurgical bond. Other factors, such as the involvement of challenging reinforcement phase and a wide selection of base metal that forms the matrix phase, can significantly improve the cladding layer properties [14,15,16,17,18,19].

**Figure 4:**
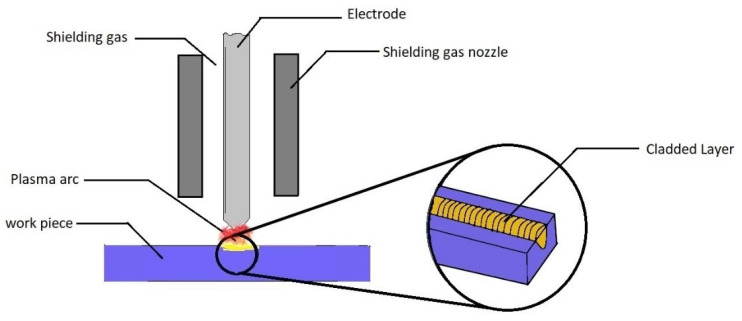
Schematics of gas tungsten arc welding (GTAW).

One of the methods to apply GTAW starts with preparing the cladding mixture. Further, this can be done by mixing the required ceramic powder with a base powder to which steel balls can be added for uniformity in the mixture. These materials are then filled and rolled into a strip of appropriate size with a low or medium carbon steel tube. After this, the strip is fixed to any required substrate on which coating has to be performed. GTAW can then be performed on the above system with suitable welding parameters [20,21]. Figure 4 depicts the above method schematics.

The second and the recently developed method is the in situ production of ceramic coating on the substrate assisted by a chemical reaction. The in situ development helps remove any impurity and interfacial incompatibility of the coating powders with the substrate, resulting in stronger metallurgical bonds and improved mechanical characteristics. The cladding powder is prepared by mixing appropriate reactants in a suitable stoichiometric ratio favoring a chemical reaction with the substrate material to produce the required ceramic coating [22]. Coatings with multiple ceramic particles can also be added further to enhance the properties of the substrate [23]. The powder is blended and dried before placing it on the substrate. GTAW is then performed on this system in a single [24] or multiple pass fashion (for large scale applications) [25] with suitable welding parameters. Table 1 illustrates a summary of investigations about this process taken into account for this review paper.

#### 2.1.2. Laser Beam Cladding

**Figure 5:**
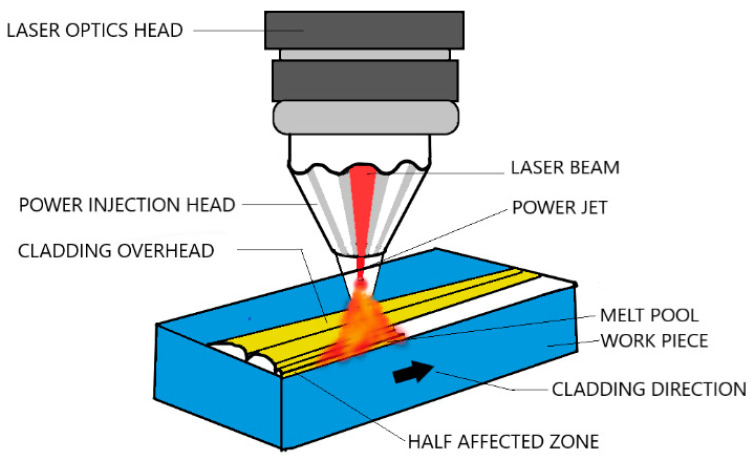
Laser cladding.

This process offers superb accuracy in a material deposition with less heat input as compared to its counterparts. The heat is supplied by a laser source on a highly concentrated zone where the cladding powder is kept on the substrate’s surface, as shown in Figure 5. The high temperatures promote the melting of the mixture with the substrate, melted by thermal conduction through the pre-placed layer [26,27,28,29,30,31,32]. Due to the interaction with the cold substrate, rapid solidification occurs, which improves mechanical properties. These factors lead to strong metallurgical bonds between the coating and the substrate as both are melted in the concentrated zone where the laser is focused. A high powder diode laser is utilized as a laser source for a wide area of applications, mainly aerospace, gas turbine, and automobile industry, where high precision is required. It can be highly automated with robot arms’ help and proved to be beneficial for large scale productions.

Another approach to this process is on similar lines as that of the GTAWs method. In situ development of ceramic particles can also be performed to ensure pure cladding particles present in the coating, thereby promoting mechanical properties. However, sometimes the chemical reaction taking place can be highly exothermic. Such processes usually lead to thermite conditions and need an external agent to slow down an exothermic reaction rate. The substrate can also be subjected to water-cooling to ensure rapid solidification. Table 2 depicts the list of studies taken into account to conclude with the cladding layer’s properties obtained through this process.

#### 2.1.3. Plasma Transferred Arc Cladding

**Figure 6:**
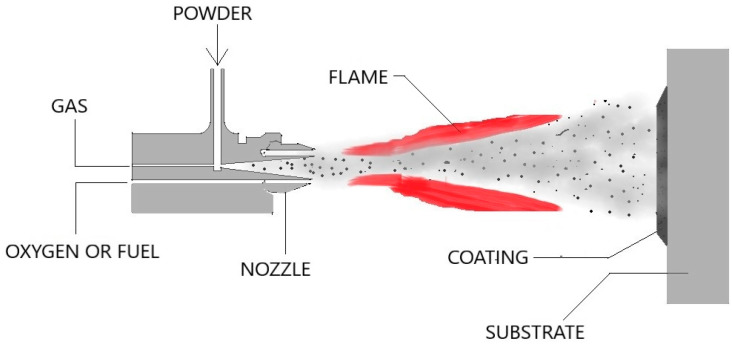
Plasma transferred arc (PTA) Process.

This is a type of thermal spraying process offering high deposition rates, cost-effectiveness, ease of operation, and a wide range of adaptability [33,34,35]. In this process, the particles are melted and then accelerated with the help of high-velocity gases. Plasma serves as the heat source, with the arc being created between the poles developed at the electrode and the nozzle. The gases usually comprise the inert gas to provide a shielding atmosphere for the cladding particles and the melting substrate. The wholly or partially melted accelerated particles solidify on impact with the substrate surface [19,36,37]. A typical Plasma Transferred Arc (PTA) cladding process is being shown in Figure 6. The obtained layer is free of any distortion and has enhanced hardness and wear resistance properties.

PTA cladding can also be utilized as a post-treatment method to Atmospheric Plasma Spraying for eliminating certain defects such as porosity, cracks, etc. from the cladding layer [38].

#### 2.1.4. Friction Cladding

Friction Cladding is an energy-efficient and eco-friendly solid-state process. A method representing friction welding utilizes thermal and mechanical methods for cladding particles onto the substrate’s surface. Independent of the operator’s skill, this method offers a broad region of operating positions and produces a high-quality coating with excellent mechanical properties [39]. It is extremely useful in joining and cladding different elements and can be used in a broad spectrum of applications.

**Figure 7:**
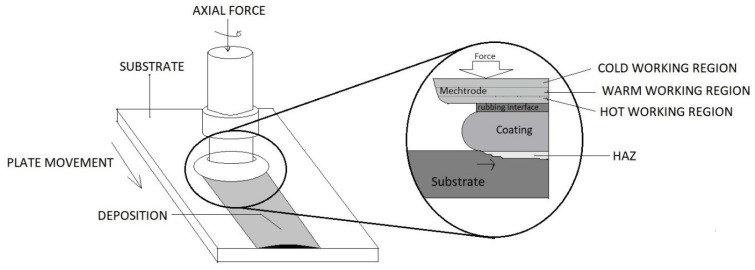
Schematics of friction surfacing.

The process includes a consumable rotating rod that experiences an axial load to press against the substrate’s surface. Further, this causes heat generation due to friction, which causes the surface to become viscous under plastic deformation. A metallic bond is formed between the coating and the substrate due to the pressure and temperature conditions. As the rotating rod moves forward, it leads to the development of continuous coating. The temperatures remain below the fusion point of both the coating and the substrate elements, and the bonding and cladding are brought only with the help of friction and plastic deformation [40]. A detailed schematic diagram is represented in Figure 7. The process parameters generally include rotation speed, travel speed, axial pressure, and feed rate. This process’s primary advantages over the other fusion methods are that as it does not go above the fusion point, no fumes/splashes occur, leading to negligible dilution. The HAZ is also highly concentrated, leading to the efficient utilization of energy [41].

A second approach developed for this process has implemented the use of the cylindrical metallic brush. This metallic brush is rotated at very high speeds while simultaneously pressing against the substrate and rod of coating material at two different places. The brush’s wires cause removing particles from the rod containing the coating particles and depositing it on the substrate surface. The coating’s uniformity is brought by the high rotation speeds and many wires present in the brush [42].

#### 2.1.5. Explosion Cladding

**Figure 8:**
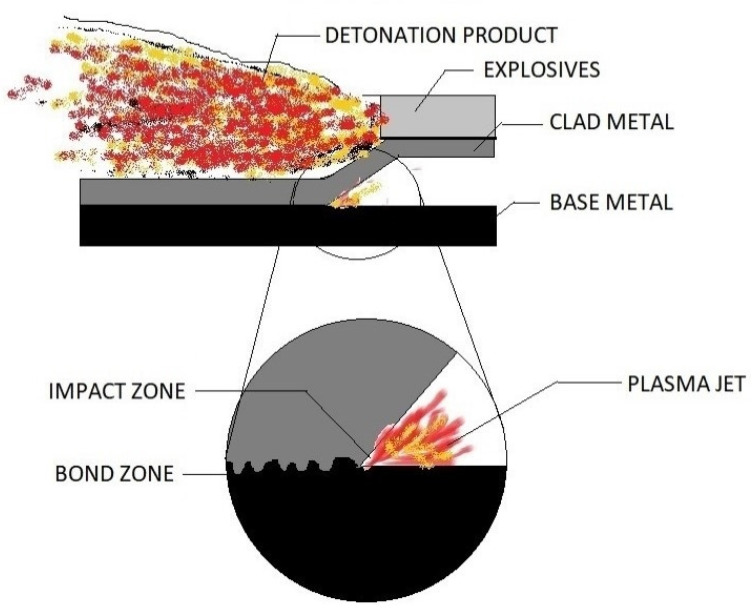
Explosion cladding.

Another solid-state method highly useful in welding/cladding different elements cannot be cladded by any other methods [43,44,45,46,47,48] and results in a dependable and robust bond. It includes chemical reactions that promote explosive detonations at very high velocity in a controlled manner to force the substrate and the coating together at elevated pressures temperatures [49,50]. The angle and velocity of collision should be within the range favorable for metallurgical bonding, promoting a high-velocity forward jet. The pressure generated is distributed so that the surfaces after colliding flows hydrodynamically. The liquid metal jet is expelled at a velocity exceeding that of the collision zone from the apex of angled collision [51]. The powder on the substrate is compressed in a radial direction as the shock wave propagates along the substrate, as shown in Figure 8. The cladding is brought about with the help of inter-atomic attraction, and the inter-locking brought about by mechanical means. The main process parameters include the composition and geometry of the materials, explosive reactants, height of explosives, arrangement type, collision angle, velocity, and energy required for bonding [39].

It has various superior qualities over other cladding methods. It produces a wavy interface with a large contact area without any intermetallic compounds present in the microstructure, which possess enhanced mechanical properties than the substrate element [52]. The HAZ is almost insignificant in explosive welding [51] due to the less time-space for melting and re-solidification. The process is found economical due to simple arrangements and shorter periods [53].

#### 2.1.6. Accumulative Roll Bonding

**Figure 9:**
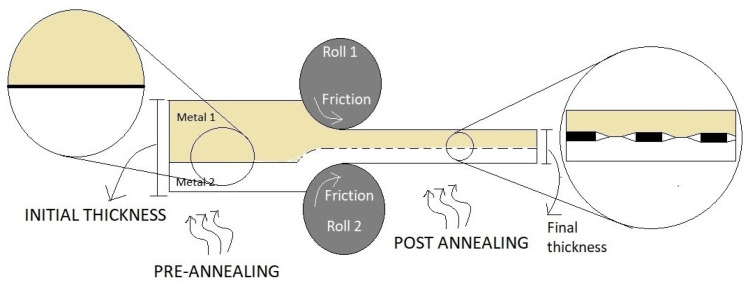
Schematics of ARB.

**Figure 10:**
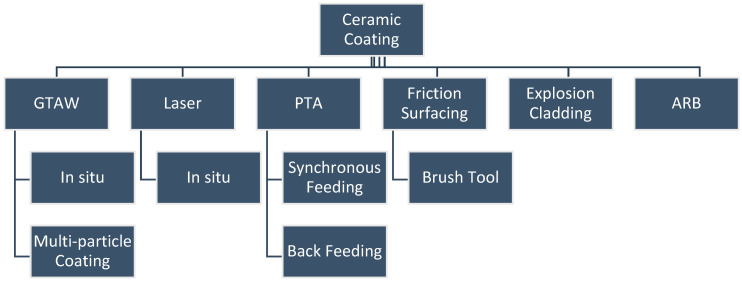
Processes and sub-systems for ceramic coating.

**Figure 11:**
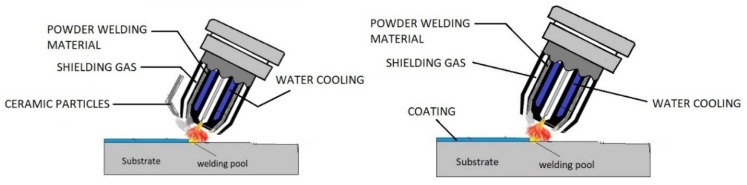
Synchronous Feeding (right) and Back Feeding (left) mechanisms.

Accumulative roll bonding or ARB is similar to the conventional roll welding process. The materials to be cladded are cut. They undergo surface treatment (degreased and brushed by wire) to promote bond formation and increase the bonding strength while removing the surface contaminants [54]. They are then stacked and rolled in a repetitive cycle shown in Figure 9. The entire process can be conducted at ambient temperature or high temperatures but below the materials’ fusion point depending upon the materials to be cladded [55]. The rolling strain is determined by the bonding, which is to be brought between the layer and the substrate [56]. The deformation is mainly brought about by plastic strain, which causes the coating layer’s cladding onto the substrate without any significant increase in the substrate [57]. The virgin material under plasticization fills up the plastic strains’ cracks, which causes the contact and bonding between the two materials [58]. The ceramic coating sequence of the flow chart is given in Figure 10. The feeding mechanism is categorized into two types, namely synchronous and back feeding systems shown in Figure 11, depending on the zone in which the cladding mixture is fed. Table 3 summarizes the experimental value of this process, taken into account for this review paper. Table 4 and Table 5 illustrate a summary of plasma cladding and explosion cladding of the observed value.

## 3. Results and Discussion

In the GTAW process, the cladding layer’s hardness was greatly affected by the welding parameters, namely welding speed and current [59]. While both the properties had a linear relationship with the above-stated welding parameters, it was noted that hardness increased while the wear resistance decreased on the increment of variables. Moreover, this was because higher heat input caused a better mixing of cladding powders and the substrate. In comparison, lower heat input promoted uniformity in the microstructure of the cladding layer. The martensitic phase occurred due to rapid solidification in the GTAW process and further strengthened the cladding layer. It was also found that the wear resistance was significantly affected by cladding powder composition and increased with the increase in hard particle concentration at a constant heat input due to dispersion strengthening within the matrix. These reinforcement particles are present in specific structures (dendritic, pellet-shaped, vein-shaped) [20], uniformly distributed within the matrix. As the bonding between matrix and reinforcement determine significant coating properties [82,83,84], the presence of dendritic patterns in the ferrous matrix caused good mechanical interlocking and contributed to enhanced hardness and wear resistance properties. Besides adding strength to the structure, the dendrites also limited plastic flow, which controlled the wear scarring at high sliding speeds.

A gradient distribution of hardness increasing from the coating-substrate interface is usually found in the cladding layer due to the difference in the ceramic particles’ densities and the substrate element, causing most of the reinforcement particles to float and finally solidify on the top [21]. The cladding layer’s in situ production provided satisfactory results. They were free from any welding defects that included cracks, inclusions, and gas porosity, but this only implied until the pre-placed layer was kept below a certain height [24]. Though the hardness increased on increasing the pre-placed layer’s thickness, welding defects simultaneously reduced the strength. Multi-particle in situ production of the ceramic cladding layer gave the highest hardness (around 1578 HV_0.2_ for [61]) and maximum wear performance while simultaneously reducing hardness gradient distribution. The overlapping passes also degraded the hardness and wear resistance properties compared to single-pass regions [25]. The properties depended on the number of deposition layers and increased only up to a critical number beyond which it showed degraded properties [60]. Further, this was highly dependent on the chemical reaction, which favored the formation of the respective cladding layer.

Laser cladding processes promoted the development of advanced coatings on the substrate. The first study in Table 2 found that the microstructures contained inter-connected plate structures (rich in Al_2_O_3_) and isolated spherical structures (rich in CaF_2_) in the inter-plate region, which also concluded that no decomposition of CaF_2_ occurred during the melting and solidification process. CaF_2_ present was found to be in the self-lubrication phase because of phase separation of the two particles about their immiscibility. The cladded layer showed enhanced wear resistance properties with a low friction coefficient under a wide range of temperature conditions due to the ceramic matrix composite layer’s low thermal conductivity. The wear resistance of the cladding layer obtained was much more than that obtained from the GTAW process and therefore showed applications in high-temperature processes. The wear resistance of metal matrix composite coatings was less than that of ceramic matrix composite coatings, as was revealed from the second case in Table 2.

Some coatings reported an increment of wear resistance as the temperature was increased until a certain point beyond which they experienced severe plastic deformations and softening [63]. The coating’s hardness increased linearly with the increase in reinforcement particles in the matrix to an extent beyond which it started decreasing. Moreover, this was due to large pores and a decrease in the metallurgical bond’s strength at a high volume percentage of ceramic particles. M.J. Tobar et al. found that on coating NiCrBSi-WC on stainless steel substrate, the best results were obtained at 25 wt %. In comparison, the results significantly decreased after 50 wt % of WC content [64]. The increase in hardness and wear resistance at the high temperature of NiCrBSi-WC coating compared to NiCrBSi due to hard particles, WC, and WC2 were uniformly distributed within the matrix to provide strength [32].

**Figure 12:**
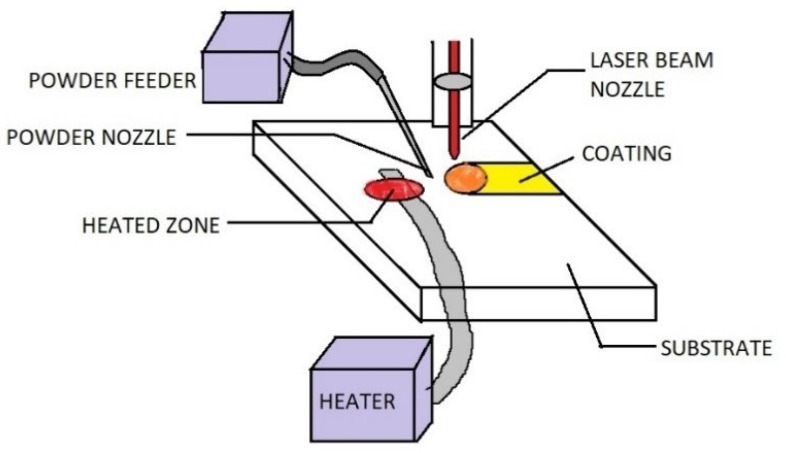
Pre-heating surface treatment.

The hardness is also dependent on the laser strength and increases with an increase in laser power. Further, this continues to some extent mainly around 2.0 kW for a laser velocity rate of 0.5 m/s for aluminum alloys [62,85] post, which the surface tends to become rough non-homogenous on solidification, which increases the wear rate and degrades mechanical properties of the cladding layer. In situ production promoted improved hardness and wear resistance properties with an average highest value of 1250 HV_0.2_ [65]. Moreover, this could be enhanced by using the multi-particle system, as discussed in the GTAW process. The distribution of TiC particles and hence the hardness was highly dependent on the laser velocity. For velocities above 8 mm/s (critical velocity), trapped TiC particles were leading to uneven distribution, which finally compromised hardness in some coating regions. However, velocities can be increased significantly with increased deposition rates by preheating the substrate [66] to reduce the cladding time. The water-cooled laser-induced thermite process in case 7 in Table 2 promoted delicate dendrite structures resulting in a maximum average hardness ranging from HV_0.2_ 2300–3060, which was more significant than any other obtained from GTAW, laser, and plasma coating process. Pre-heating surface treatment (two-step), as shown in Figure 12, further improved the hardness and wear resistance properties of the coating.

In plasma cladding, similar dendritic structures were found in the above two processes, and the cladding layer possessed excellent hardness and wear resistance properties. The hardness was uniformly distributed except for the coating’s lowermost region, showing a significant drop in hardness. The worn surface was smooth, and the wear resistance was 35 times higher, with a reduction in friction coefficient by as much as 40% compared to the pure substrate material [86]. Porosity, splat interfaces, and inter/intra cracks appeared in the coating microstructure due to substantial temperature differences. It decreased solidification time, leading to a decrease in performance in the wear and hardness test. The defects were dependent on the cladding particles; therefore, the coating layer’s judicious selection is essential in this process. The defects, mainly the cracks, propagated along the boundaries and heavily downgraded the wear performance [33]. However, the impact of inhomogeneity in the material properties can be reduced by implementing a gradient cladding layer (addition of one more layer) over the substrate [72]. Moreover, this additional layer can remove the porosity and other defects to too much extent and remove the sudden dip in hardness towards the coating-substrate interface. Laser re-melting can also be preferred as a post-process surface treatment to remove the porosity and improve hardness (up to 85% in some cases) and wear resistance capabilities of the coating [34]. Certain coatings also possessed excellent wear resistance at temperatures above 500 °C [69], thereby utilizing high-temperature applications [87]. Guoliang Hou et al. found that the aesthetic quality of coating, efficiency of the process, and the cladding layer’s mechanical properties were highly dependent on the primary gas flow rate [35]. While lower flow rates caused complete melting of the particles but decreased the impact velocity and hence the area covered during the spray, higher flow rates caused partial melting and higher impact velocity. Most of the particles tend to rebound off from the surface of the substrate.

**Figure 13:**
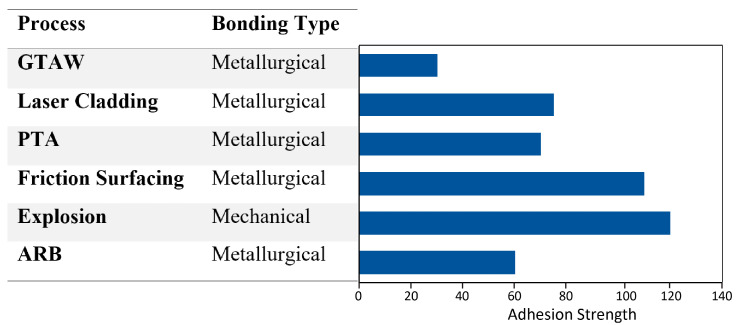
Bonding type and adhesion.

Moreover, the partially melted particles tend to experience longer cooling rates, causing an amorphous state in the microstructure. The low and very high gas flow rate samples also suffered from porosity due to the particles’ inadequate compaction during solidification. The fact that almost all the mechanical properties of the cladding layer depend upon the splats [68,88,89]. It can be concluded that an optimal velocity is necessary that could be just enough to completely melt the particles while providing sufficient kinetic energy to form efficient splats on the surface to ensure excellent post-cladding performance. Back feeding mechanism can also be preferred over the synchronous feeding mechanism to improve the coating’s hardness and wear resistance properties [90]. However, in an investigation by Ghadami et al., it was found that the properties of surface melted coatings such as laser cladding, etc. were much better than the sprayed coatings such as plasma cladding [91]. Figure 13 depicts the adhesion structure and bonding mechanism of all the processes.

The coating obtained with friction surfacing was free of any discontinuities without any cracks. It even performed well in the most difficult bending test and showed no signs of delamination. The boundary line, though observed, showed no sign of cracks and discontinuities [92]. The ceramic particles were uniformly distributed within the cladding layer, even close to the coating-substrate interface [42], which is not the case of fusion coating methods. Low yield strength particles in the coating and ceramic particles can also decrease residual stresses, resulting in a crack-free cladding layer [93]. There was, however, the presence of atmospheric gases like oxygen and nitrogen in the microstructure, mainly at the grain edges and boundaries. Moreover, this downgraded the excellent wettability property of pure titanium due to the formation of oxides and nitrides [94] in the layer leading to inhomogeneity. Further, this could be prevented by using the inert atmosphere during the process, which becomes a significant factor for strong bonding and the coating’s excellent mechanical properties. There was an interdiffusion of cladding particles into the substrate, resulting from a strong metallurgical bond between the two [95].

If a separate feeding mechanism is provided along with the consumable rod, then the cladding powder should be fed at a quarter radius offset due to various reasons. If the powder is fed at the center, it will scratch out the material, while if it is fed near the circumference, it will be carried along with the mass flow to form flash [96]. More ceramic particles could be fed by increasing the number of feeding points and the consumable rod’s suggested locations, but it tends to increase the process temperature. Though ceramic particles can usually bear such high temperatures, they can suffer from oxidation due to atmospheric gases around the cladding region. However, the cladding layer’s thickness seems to decrease, along with the cladding particles’ increase in the layer. The grains formed are ultra-fine with this process’s help, leading to a significant enhancement of mechanical properties, especially the substrate’s hardness and wear resistance.

**Figure 14:**
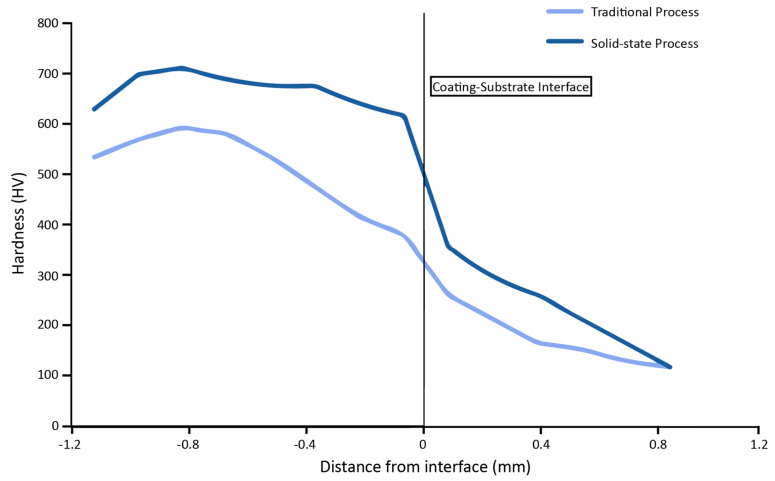
Distribution of hardness along with the depth of a sample.

In explosion cladding, the sudden increase in temperature and pressure resulted in the intense fracturing of original grains present before the cladding process. They caused the development of much finer grains post cladding leading to denser coatings [77,80,97,98]. Cracks and micropores were familiar in the microstructures due to the intense shock wave and the ceramics’ brittle nature [74,79]. However, these can be reduced by adding certain particles that can compensate for ceramics’ brittle nature. A.G. Mammalis et al. found that the peak shock significantly affected the properties of the cladding layer. A lower shock value leads to inadequate compaction, while a high value caused amorphous regions within the layer [73]. No significant deformation of the base substrate was found after the explosive detonation [75,78]. Lalu Gladson Robin et al. reported that the coating layers cladded with the help of explosion cladding possess excellent hardness even at the coating-substrate interface [76]. A comparative analysis of hardness distribution is shown in Figure 14, which depicts solid-state cladded coatings’ superiority over those cladded by traditional processes. On increasing the loading ratio, there is a transition of bond interface to a wavy pattern, which increases the mechanical interlocking and consequently increases the hardness and wear resistance of the coating [81,99,100]. Raghukandan et al. found that wire mesh presence promotes the detonation propagation and absorption of excess energy [101]. The maximum temperatures were found in the top layer, which reduced towards the bottom due to the ceramic particles’ low thermal conductivity [102].

**Figure 15:**
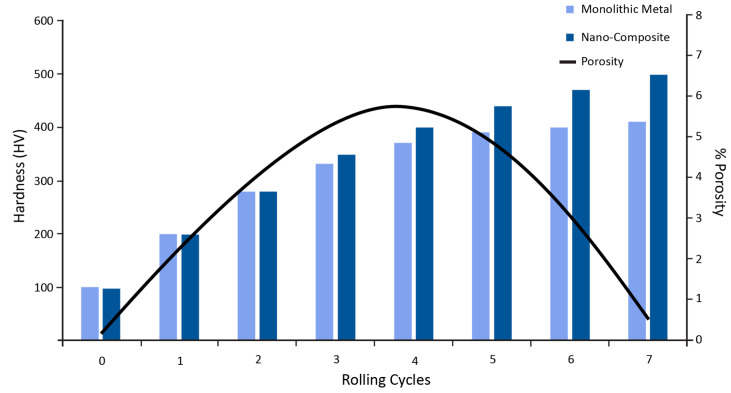
Hardness and porosity dependence on rolling cycles.

In roll bonding, successful grain refinement, and removal of initial porosity and clustering is brought during the process after repeated cycles [103,104]. The ceramic particles have a strong metallurgical bond between the coating and the substrate without many clusters and particles free zones increasing microhardness [105,106]. After a particular cycle, there was not much improvement in the microhardness, but an increase in shear strength was observed [107]. Since there is a large free surface area, it is generally possible for the particles to get oxidized. Hence, oxide particles are present in abundance in the microstructure [108]. The distribution of challenging phase particles takes place in rolling and standard directions [109]. Porosity is present in the cladding layer, which decreases the density of the coating layer. However, it starts decreasing after a certain number of cycles with the enhancement of bonding quality, as shown in Figure 15.

The coated layer’s toughness was enhanced with the increase in the rolling strain due to the elongation until the maximum stress between particles is reached. A significant advantage of this process is that no reaction occurs between the coating particles and the substrate. Microhardness can still be increased by implementing cross accumulative roll bonding (CARB) process, which involves the rotation of strips to 90° about the normal axis after each rolling cycle, causing the sheet elongation to occur in both directions. This process increases tensile strength and uniformity compared to the conventional roll bonding method and hence is proven better than that [110,111]. However, individual particles’ addition leads to a reduction in bond strength, fracture toughness of the coating, and decreased overall welding efficiency [112]. The deformation required for adequate bonding was slightly significant, but it can be reduced by increasing the temperature during the rolling process.

Traditional welding methods are capable of coating thick layers of ceramic particles onto the substrate, but the characteristics of coating obtained is subsequently poor as compared to solid-state processes in terms of hardness and wear resistance. The higher operating temperatures of processes like GTAW, Laser cladding, and PTA leads to greater percentages of dilution of substrate material into the coating particles which causes the deterioration of the ceramic characteristics. GTAW produces the thickest layer but defects like porosity are much more prevalent than its counterparts in traditional processes. Laser cladding and PTA processes have excellent overlaying capabilities as compared to GTAW and provide good control over the process parameters. Laser cladding also has the least amount of heat input out of the three, leading to a small HAZ which prevents the deterioration of ceramic characteristics, thereby producing supreme quality coatings among the traditional processes and can be fully automated to produce consistent coatings at a faster pace.

**Figure 16:**
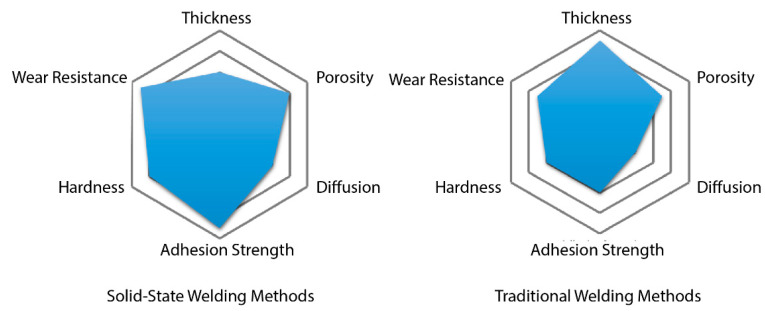
Property overview of methods.

Solid-state processes on the other hand remains below the fusion points and are much more resistant to welding defects. The only disadvantage is the requirement of high cost equipment and the slower transverse speeds of these techniques. Explosion cladding is one of the cheapest among these methods and has a simple approach to clad large surfaces without requiring much of pre-preparation of the substrate. Friction stir welding is the most versatile method with superior cladding properties while also being energy efficient. ARB, on the other hand, requires a longer operating time due to repetitive cycles so as to produce reliable coatings comparable to other methods. Table 6 denotes the technical specifications of properties of the respective processes, and Figure 16 gives a brief overview of the capabilities of the two broad categories. It clearly shows that the solid-state processes are more superior to the fusion processes with more refined microstructures and enhanced properties, including but not limited to hardness, wear and abrasion resistance, coating strength, etc.

## 4. Conclusions

Several papers were considered for investigating traditional and solid-state processes, respectively. Numerous processes and sub-processes or sub-systems were analyzed based on the experimental or computationally calculated numerical data available to the present. The processes were tested based on microstructure, hardness, wear, and abrasion strength, respectively. The defects were analyzed, and solutions were also considered for their potential in this comparative analysis.

- Solid-state processes did not face any potential challenges due to the dissimilarity in the ceramics and metals properties. The temperature remained below the fusion points of both the materials, and hence factors such as thermal expansion did not affect the quality of the cladding layer.
- As the fusion welding processes involved melting and re-solidification, porosity could be seen in the microstructures unless an adequate inert environment is provided during the cladding process. The solid-state processes, on the other hand, faced comparatively less porosity and hence had better properties.
- The hardness was uniformly distributed in the entire coating in solid-state cladding processes, while fusion welding processes suffered a gradient decrease in hardness to the coating-substrate interface.
- The overall hardness of solid-state processes was also found on the higher end, as the comparative study was considered between the two. Further, this can only be attributed to the temperatures which remain well below the fusion points.
- Solid-state processes also led to more enhancement of wear resistance properties because of the high adhesive strength provided during the process. The coatings were denser due to finer grains, bonds were more vital, and a decrease in porosity led to superior properties than the traditional fusion welding processes.

## Figures and Tables

**Table 1:** Summary of Gas Tungsten Arc Welding process parameters.

S. No.	Coating	Substrate	Operating Parameters	Electrode	Shielding Gas	Ref
1	SiC	S50C Steel	Current: 155/140/120 A Voltage: 17/20 V Speed: 8/11/14 cm/min	Thorium (DCSP)	Argon (8 L/min)	[59]
2	WC-Ti	Steel	Current: 100 A Speed: 13 cm/s	Thorium (DCEN)	Argon (8 L/min)	[20]
3	TiC	Carbon Steel	Current: 150 A Speed: 10 cm/min	Thorium	Argon (8 L/min)	[21]
4	TiC	AISI 1045 Steel	Current: 150 A Voltage: 15–17 V Speed: 5.5 cm/min	Thorium (DCSP)	Argon (8 L/min)	[22]
5	Fe-TiC	AISI 1045 Steel	Current: 90–180 A Voltage: 15–17 V Speed: 7.0–8.5 cm/min	Tungsten (DCSP)	Argon (8 L/min)	[24]
6	TiC/Ti_5_Si_3_	Ti-5Al-2.5Sn	Current: 300 A Voltage: 20 V Speed: 1.5 mm/s	-	Argon (0.3 L/s)	[60]
7	TiB_2_-TiC-Al_2_O_3_	AISI 1020 Steel	Current: 100 A Voltage: 15–17 V Speed: 1.5–2 mm/s	-	Argon (10 L/min)	[23]
8	WC	Q235 Steel	Current: 150 A Voltage: 16–18 V	Tungsten	Argon (8 L/min)	[61]

**Table 2:** Summary of Laser Cladding parameters.

S. No.	Coating	Substrate	Laser Power (kW)	Beam Scanning Speed	Ref
1	CaF_2_/Al_2_O_3_	Al_2_O_3_	2.0	60 mm/min	[62]
2	WC/TiC	Al Alloy	2.0	-	[31]
3	Ni/hBN	Stainless Steel	1.0–3.6	70–500 mm/min	[63]
4	NiCrBSi-WC	AISI 304 Steel	0.5–2.25	100–110 mm/min	[64]
5	TiC	Stainless Steel	2.0	4–15 mm/s	[65]
6	WC	A3 mild Steel	5.0	2000–3000 mm/min	[66]
7	Al_2_O_3_	AA7075 Aluminium	1.8	1500 mm/min	[67]

**Table 3:** Roll cladding procedure.

S. No	Coating	Base Metal	Roller Speed	Temperature (K)
1	Al	Cu	1.5(m/min)	398
2	Al	Cu	2(m/min)	773
3	B_4_C and SiC	Al	-	-
4	Al	Al_2_O_3_	5 rpm	993
5	Al_2_O_3_ and SiC	AA1100	5 rpm	473

**Table 4:** Plasma cladding procedure.

S. No.	Coating	Current(A)	Voltage(V)	Ar Gas Flow Rate(L/min)	H_2_ Gas Flow Rate(L/min)	Carrier Gas Flow Rate(L/min)	Spray Distance	Ref
1	YSZ	450	55	42	8	480	20	[68]
2	Cr_3_C_2_-Ni	600	50	42	9	480	20	[69]
3	Al_2_O_3_	660	50	45	9	400	10	[33]
4	Cr_2_O_3_	620	50	45	12	400	10	[33]
5	Zr_2_O_3_	700	55	42	8	400	15	[68]
6	Mo	500	65	27	15	3–6	10	[70]
7	Al_2_O_3_	500	70	50	15	2–6	10	[71]
8	3Al_2_O_3_∙2SiO_2_	450	50	35	0.5	8	10	[35]
9	Cr_3_C_2_	100	30	15	1.7	2	8	[72]

**Table 5:** Experimental values of Explosion Cladding.

S No.	Dimensions of the Flyer Plate, mm	Dimensions of the Parent Plate, mm	Type of Explosive	Detonation Velocity, Lid	Initial Set-Up Angle	Collision Angle	Stand-Off Distance	The Thickness of the Explosive He	Impact Mass Ratio, R	The Velocity of the Flyer Plate, V	Peak Shock Pressure, P	Ref
1	200 × 40 × 2	200 × 40 × 2	Paxit	4000	0	13	2	25	0.6	1047	10	[73]
2	90 × 30 × 2	200 × 40 × 5	Nitrammite	4000	0	15	2	35	1.2	1286	11	[74]
3	210 × 30 × 1.5	187 × 38 × 1.5	Paxit	4000	0	12	2	30	0.9	2200	8.6	[75]
4	80 × 60 × 2	80 × 60 × 5	SUN90	4500	0	0	3.5	28	0.9	1500	13	[76]
5	215 × 70 × 5	210 × 60 × 4	Nitrammite	4000	6	16.1	4	40	1.05	1780	14	[77]
6	100 × 100 × 4	Ø100 × 36	Amonit	4500	0	10	4	25	0.8	1100	7	[78]
7	165×70×1.5	100 × 55 × 6	Amonit	4500	0	13	4	30	0.85	2000	6	[78]
8	200 × 50 × 3	180 × 40 × 3	Paxit	4000	0	15	2	2.5	0.7	1800	5	[79]
9	Φ19.05 (OD) × 1.65 (Wall) × 240	Φ13.75 × 240	Nitrammite	4000	0	0	3	11	0.9	1700	5.2	[80]
10	80 × 60 × 2	80 × 60 × 5	SUN 90	4500	0	14	6.5	25	0.75	2100	7	[81]

**Table 6:** Property comparison [113,114,115].

Process	Max. Coating Thickness Obtained (mm)	Dilution	Porosity
GTAW	0.2–10	5–15%	2–8%
Laser Beam	0.2–2	<5%	<0.1%
PTA	0.5–5	5–15%	<0.5–2%
Friction Stir	0.5–3	<3%	0.5–1%
Explosion	0.1–0.5	<3%	<1%
ARB	1.5–5	5–15%	1–5%
